# Ultrasound measurement of optic nerve sheath diameter pre and post lumbar puncture for prediction of postdural puncture headache

**DOI:** 10.1038/s41598-026-40311-1

**Published:** 2026-02-20

**Authors:** Fatma Merzou, Anna-Lena Kunzmann, Daniel Janitschke, Jose Valdueza, Benjamin Landau, Sebastian Roemer, Erwin Stolz, Laurin Schappe, Viviana Versace, Steffen Kottackal, Piergiorgio Lochner

**Affiliations:** 1 Department of Neurology, Saarland University Medical Center, Homburg, Germany; 2 Neurological Center, Segeberger Kliniken, Bad Segeberg, Germany; 3 Department of Neurology, LMU University Hospital, LMU Munich, Munich, Germany; 4 Medical Faculty, Justus-Liebig-University, Gießen, Germany; 5 Department of Neurorehabilitation, Hospital of Vipiteno (SABES-ASDAA), Vipiteno-Sterzing, Italy; 6 Department of Neurology, Saarland University, Campus Homburg, Building 90, Kirrberger Straße, 66421 Homburg, Germany

**Keywords:** Optic nerve sheath diameter, Transorbital sonography, Lumbar puncture, Postdural-puncture headache, Intracranial pressure, Spontaneous intracranial hypotension, Diseases, Health care, Medical research, Neurology, Neuroscience, Signs and symptoms

## Abstract

The aim of our study is to test the hypothesis whether ultrasonographically measured ultrasound-guided optic nerve sheath diameter (US-ONSD) decreases after lumbar puncture (LP) and whether decreased optic nerve sheath diameter (ONSD) after lumbar puncture is associated with headache. The latter might help to identify patients with postdural puncture headache (PDPH). In this prospective observational study 76 patients, who had undergone diagnostic LP using an atraumatic technique, received a measurement of ONSD before (T0), immediately after (T1) and 24 h after LP (T2). Additionally demographic data such as age, sex, body mass index (BMI), and also headaches and symptoms were recorded. In six out of 7 patients with constant PDPH, we additionally measured ONSD 48 h (T3) and 72 h (T4) after LP. All patients (n = 76, 100%) showed a physiological reduction in ONSD at T1. Patients with consistent symptoms of PDPH (n = 7, 9%) kept values below pre-LP levels after 24 and 48 h. No statistical difference was found in BMI, sex, cerebrospinal fluid volume, needle size, or previous headaches between the PDPH (n = 7, 9%) and non-PDPH patients (n = 69, 91%). Younger patients were more likely to experience PDPH symptoms. Since at T2 the ONSD was only reduced in PDPH patients, a significant difference in ONSD was found between PDPH and non-PDPH patients. The cut-off value of ONSD for predicting PDPH at T2 was 4.9 mm in the receiver operating characteristic (ROC) curve (sensitivity 86%, specificity 93%). We were able to demonstrate a physiologic change in ONSD after LP in all patients. The sonographic measurement of ONSD in patients with headache can help to identify and monitor PDPH after LP.

**Supplementary Information:**

The online version contains supplementary material available at 10.1038/s41598-026-40311-1.

## Introduction

Postdural puncture headache (PDPH) due to a cerebrospinal fluid (CSF) leak following a dural puncture, such as the diagnostic lumbar puncture (LP), is characterised by a position-dependent headache, usually occurring within five days after the procedure and disappearing spontaneously after two weeks. Neck pain, visual disturbances, back pain, subjective hearing disturbances, and nausea may also occur. The development of post-puncture syndrome depends, among other things, on the shape and size of the needle. Predisposing factors such as young age, female sex, low body mass index (BMI), and chronic headache also play a role^1,2^.

PDPH impairs the quality of life of the affected patients and requires additional treatment, which can lead to hospitalisation and treatment costs. Optic nerve sheath diameter (ONSD) measurement via transorbital sonography (TOS) is based on the measurement of the optic nerve sheath (ONS) that surrounds the optic nerve. The optic subarachnoid space communicates with the cranial subarachnoid space, lies between the optic nerve and the ONS, and is filled with CSF. It is known that an increase or decrease of intracranial pressure (ICP) causes the ONS to increase or decrease in diameter^3^. Meanwhile, several studies have shown a correlation between the increase in the ONSD and increased ICP^4,5^. However, there is currently limited data to determine the utility of ultrasound-guided ONSD (US-ONSD) after LP; particularly whether ONSD can also specifically detect low ICP^6–8^.

In this context, according to the pathophysiology and magnetic resonance imaging (MRI) findings of symptomatic intracranial hypotension, a pilot study from Fitchner et al. showed that symptomatic patients with orthostatic headache had a significantly decreased ONSD during changes from supine to upright position^9^.

PDPH is a common complication after LP and there exists no effective tool for predicting it yet. According to a study published in 2024, ONSD may be valuable for predicting PDPH^7^.

However, to date there have been no studies looking at patients receiving LP for diagnostic purposes without an anaesthetic procedure and serially measuring ONSD to identify individuals at risk of developing PDPH^8^. There are conflicting results in the literature on the correlation between the change in real-time ONSD and the measurement of ICP by lumbar puncture, with two reports finding a positive correlation and one finding no correlation^10–12^.

### Aim

We tested the hypothesis that US-ONSD is changed after LP and could identify patients with possible occurrence of PDPH.

## Methods

### Study design

This prospective observational study was conducted in the department of neurology at the Saarland University Medical Center. The local ethics committee (Ethikkommission der Ärztekammer des Saarlandes; no. 208/23) approved this study. All procedures were performed according to the ethical standards of the committee responsible for human experimentation (institutional and national) and the Helsinki Declaration of 1975, revised in 2013. Written informed consent was obtained from each participant or legal guardian.

### Participants

**Fig. 1:**
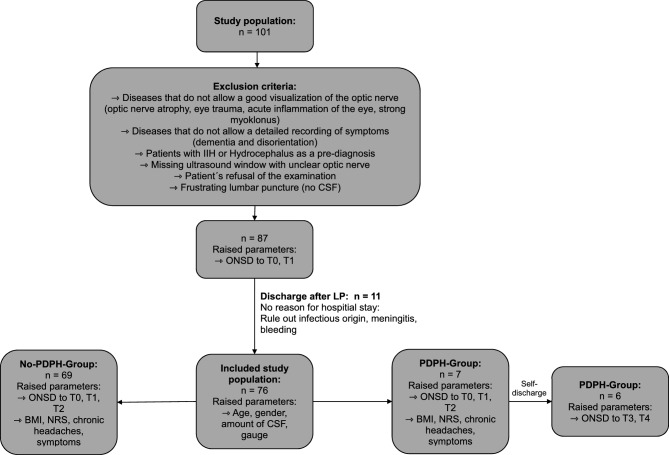
Flow chart: The clinical course of study for patients.

We prospectively recruited patients, who were admitted to our neurology department between November 2023 and April 2024 and needed to undergo LP as a diagnostic test. ONSD was measured using ultrasound in each patient before and after LP. Inclusion criteria were as follows: age ≥ 18 years, patients who require LP solely for clinical indication as diagnostic test (patients with headaches, autoimmune diseases (e.g. multiple sclerosis), infections of the central/peripheral nervous system, or unclear polyneuropathy). All patients had no clinical signs of increased ICP before LP and underwent an LP using an atraumatic needle, preferably the size 20G (n = 66, 86.8%) but also 22G (n = 6, 7.9%) and in rare 21G (n = 2, 2.6%) and 27G (n = 2, 2.6%). The exclusion criteria were age < 18 years, no informed consent, presence of absolute contraindications for LP, poor or unavailable transorbital acoustic window (e.g., due to severe optic nerve atrophy, preexisting eye trauma) and patients with hydrocephalus or idiopathic intracranial hypertension (IIH) (Fig. 1).

All patients showed no clinical signs of increased ICP and underwent a cranial computed tomography (CT) scan of the head before the LP procedure.

Patient characteristics were collected before LP. LP was performed in a seated position with both knees and chin pointing towards reaching for the chest. CSF pressure was not measured, as there was no clinical indication.

For ONSD measurement, the patient was positioned supine with the head and upper body raised 20–30°. US-ONSD technique is subsequently described in detail in the same section^13^.

**Fig. 2:**
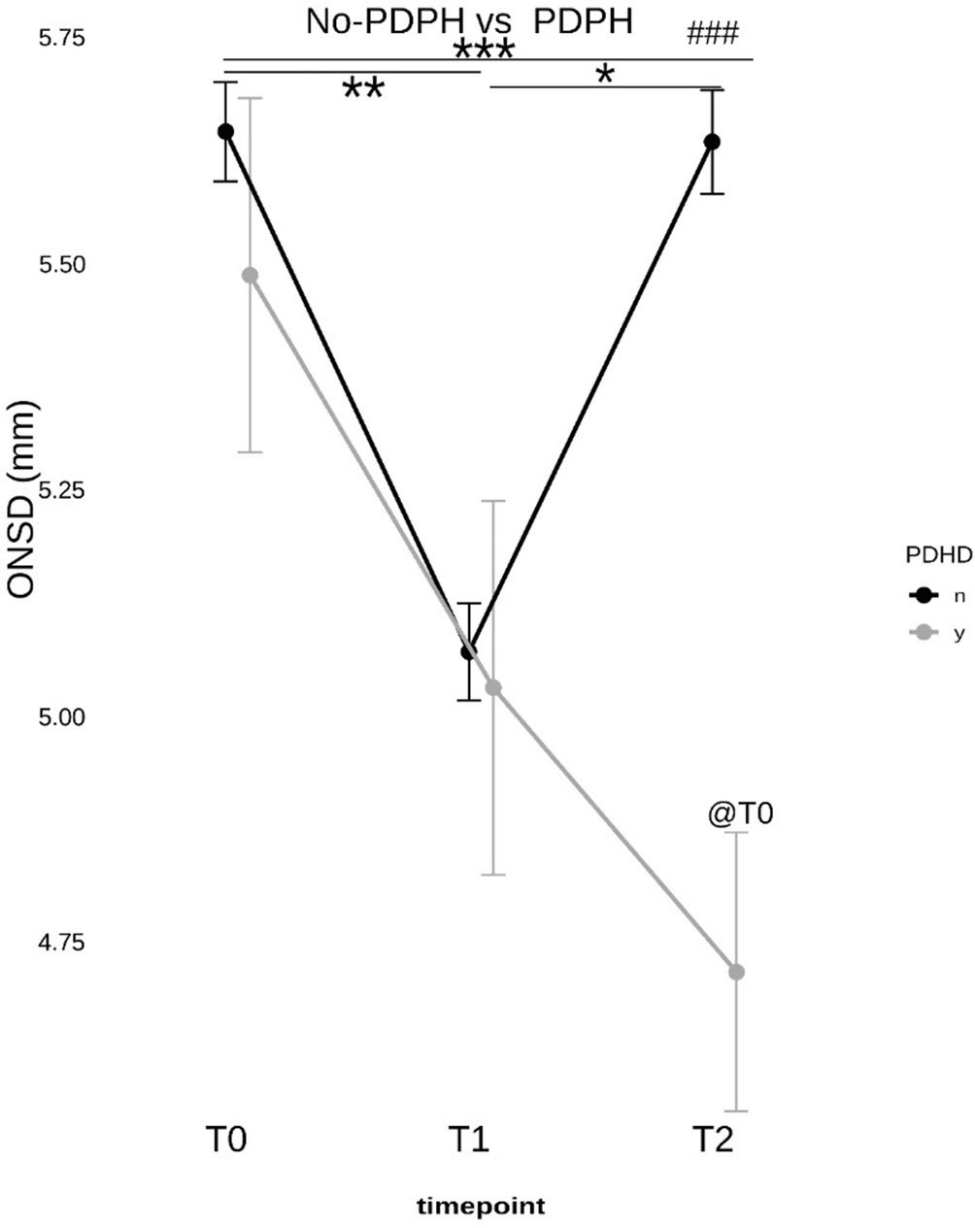
The ONSD measures values of the two groups of post-dural puncture headache (PDPH) and no PDPH at different time points. Before lumbar puncture (T0), after LP (T1) and after 24 h (T2). Asterisks denote statistical significance at * p < 0.05, ** p < 0.01, and *** p < 0.001. ### p < 0.001 compares T2 between groups. @T0 p < 0.05 compared to PDHD.

**Fig. 3:**
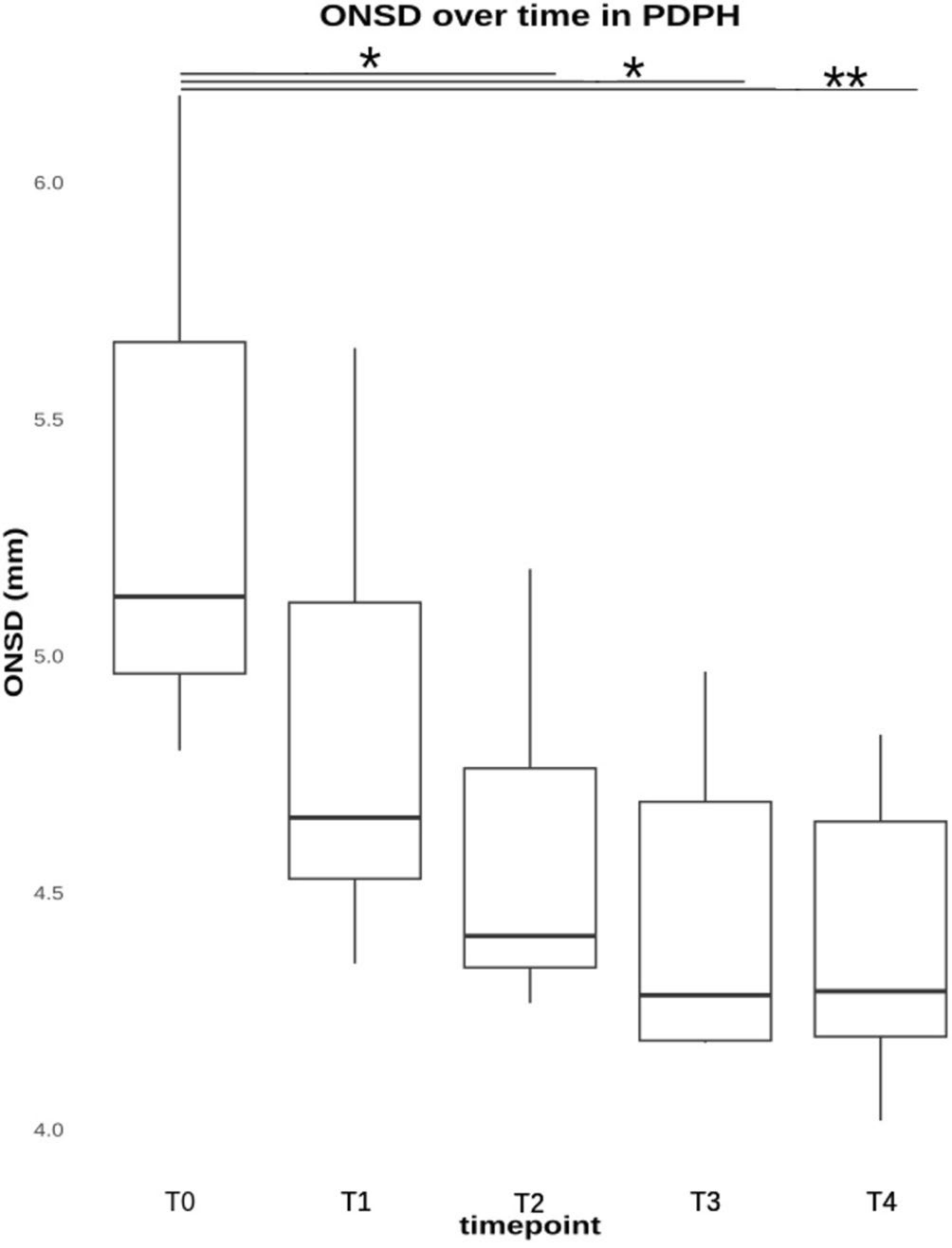
The ONSD measurement values of the patients with postdural puncture headache (PDPH) at different time points. Before lumbar puncture (T0), directly after LP (T1), after 24 h (T2), after 48 h (T3), and after 72 h (T4). * p < 0.05, ** p < 0.01, *** p < 0.001.

ONSD was assessed before LP (T0), immediately after LP (T1), and after 24 h after LP (T2) (Fig. 2). If patients presented with symptoms of PDPH related to CSF hypotension, clinical data were collected up to 72 h (T4), and ONSD was measured at T3 (48 h) and T4 (72 h) (Fig. 3).

The datasets used and analysed during the current study are available from the corresponding author upon reasonable request.

### Transorbital sonography

Before commencement of this study, the investigator (AK) was trained to perform ONSD measurement by ultrasound, examining 25 healthy subjects according to the guidelines of the recent international measurement protocol^14,15^ and was supervised by a clinician experienced in the technique (PL). Subsequently, the two sonographers (AK and PL), who investigated the inter-observer variation independently performed ONSD measurements without knowing the other’s results before starting recruitment and obtaining sonographic measurements. The average inter-observer variation was 0.13 mm.

### US-ONSD measurement technique

The ultrasonography studies of ONSD were performed with an ultrasound machine (GE Logiq E9) equipped with a linear transducer (LA 332, GE; 4–15 MHz) with a lateral resolution of < 0.4 mm; the mechanical index was reduced to < 0.26, following ALARA principle.

In particular, the ONSD was identified by measuring the distance between the external borders of the hyperechogenic area surrounding the optic nerve, corresponding to the periorbital subarachnoid space (supplemental Fig. 1). The binocular mean ONSD values were obtained for each eye by averaging these values.

Brightness and dynamic range were individually modified in order to obtain more contrast. Once we had achieved a satisfactory image of the ONSD, this was captured using the freeze button and the measurement was performed on the ultrasound device. Three different measurements were taken for each eye, and the average value was used for statistical purposes. For a standardized measurement we went 3 mm downwards starting from the lamina cribosa. The optic nerve presents itself as hypoechoic. Next to the hypoechoic optic nerve one can identify the hyperechoic ONSD according to recently published consensus criteria^15^.

All transorbital ultrasound investigations were performed by one person (AK), who was blinded to medical examinations, using the same equipment and constant settings. In cases of measurement uncertainty, the ONSD measurements were reviewed again with the senior neurosonologist (PL).

### PDPH patients

The clinical course of PDPH patients was followed up by an additional third and blinded investigator, who was not responsible for measuring the ONSD so that subjective factors influencing the study results were avoided.

The patients were questioned for the development of PDPH at the 24th, 48th or eventually at 72th hour, and the numerical rating scale (NRS) score of patients who developed PDPH was recorded. Postinterventional PDPH pain assessment was measured with an NRS score from 0 to 10 (0 = no pain and 10 = most severe pain) by patient self-assessment. Furthermore, we contacted the patients two weeks after the LP to hear more about the course of their symptoms after discharge.

Moreover, age, sex, weight, height, and correspective BMI, needle thickness and amount of CSF were also recorded for all patients. The amount of CSF, extracted during the lumbar puncture, was determined by a doctor, depending on the underlying disease and indication and ranged from a minimum of 5 to a maximum of 30 mL.

The selected patient position was in sitting, which is better tolerated by most patients in an acute setting.

### Statistical analysis

Based on previous studies, the frequency relation between the two groups PDPH and non-PDPH patients is assumed to be at approximately 1:4^7^. In order to be able to determine a significant difference between these two groups, the needed number of patients was calculated to be 80 persons altogether.

All collected variables were stored in one table. This dataset was evaluated using the programming language ”R” for statistical analysis and the software ”RStudio”. For quantitative data, descriptive statistics were reported as mean (± standard deviation (SD)) for normally distributed variables and median (min–max), if not normally distributed, respectively. Absolute frequencies and percentages were reported for qualitative variables. Student’s t-test was used for continuous variables to determine significant differences between independent groups, while the chi-squared test was employed to evaluate differences in categorical variables.

Multivariate logistic regression was performed to determine the associations of the demographic and procedural variables highlighted (sex, age, CSF volume withdrawn, needle gauge, history of chronic headache, and BMI) as well as the ONSD measured at three time points (T0 = pre-puncture, T1 = immediately post-puncture, T2 = 24 h post-puncture) and the relationship with PDPH.

The statistical evaluation of ONSD over time involved a two-factorial ANOVA followed by a Tukey-HSD as a post-hoc analysis. ROC curve analysis was conducted using the “pROC” package for R. Plots were generated using the “ggplot2” package. In all figures, asterisks denote statistical significance at * for p < 0.05, ** for p < 0.01, and *** for p < 0.001.

## Results

Altogether 101 patients were assessed. Figure 1 (flow chart) shows the algorithm for excluding and including patients, according to the specified reasons.

All 87 patients underwent an LP and a measurement of the ONSD. In 11 patients the ONSD was only recorded at T0 and T1 because they did not provide an indication for hospital stay. The reasons for exclusion are shown in the flow chart. Finally, 76 patients were included for analyses. The characteristics of patients are listed in Table 1. Seven (9.2%) of the 76 patients (5/2; f/m) developed PDPH.

**Table 1:** Demographic data of the PDPH and non-PDPH groups.

Characteristic	Non-PDPH group n = 69		PDPH group n = 7		P-value
	Number (%)	Mean ± SD Median (Min–Max)	Number (%)	Mean ± SD Median (Min–Max)	
Age (years)		56 ± 17 60 (19–88)		34 ± 12 30 (21–53)	< 0.003^b^
Sex (F/M)	31/38 (45/55)		5/2 (71/29)		0.18^a^
BMI (kg/m^2^)		25 ± 5 25 (14–37)		22 ± 4 23 (16–27)	0.18^c^
Chronic headache (yes/no)	12/57 (17/83)		2/5 (29/71)		0.47^a^
Liquor volume (ml)		9 ± 7 5 (3–35)		11 ± 8 10 (5–30)	0.18^b^
Gauge	20: n = 61 (88.4%) 21: n = 2 (2.9%) 22: n = 4 (5.8%) 27: n = 2 (2.9%)		20: n = 5 (71.4%) 22: n = 2 (28.6%)		0.19^a^

a: Chi-squared test; b: Mann–Whitney-U-test; c: Unpaired t-test.

Gauge: needle size.

No statistical differences were found in terms of BMI, sex, CSF volume, needle size, or prior history of headaches between PDPH and non-PDPH patients. There was a statistical difference in age (p < 0.001). We found no significant correlation between ONSD and the NRS scores of both groups at each time point. The only significant correlation was found in the PDPH group between NRS and ONSD at time point T1 (r = -0.44; p = 0.045).

In the PDPH group, a significant correlation was found between sex and ONSD at T0 (r = -0.78; p = 0.04), T1 (r = -0.80; p = 0.03), T2 (r = -0.82; p = 0.03), T3 (r = -0.97; p = 0.002), T4 (r = -0.95; p = 0.004) and a correlation between age and ONSD at T1 (r = 0.775; p = 0.04), T2 (r = 0.773; p = 0.04), T3 (r = 0.97; p = 0.002), T4 (r = 0.90; p = 0.015). In the PDPH group, there was a significant increase in back pain in the lumbar area (p = 0.004), once from T0 to T1 (p = 0.02) and from T0 to T2 (p = 0.003). For all other symptoms surveyed from T0 to T4, no significant difference was found.

There was no statistically significant difference before lumbar puncture (T0) in the mean ONSD measurements in patients with PDPH 5.40 mm ± 0.54 (range 4.80–6.20 mm), and without PDPH development 5.61 mm ± 0.46 (range 4.52–6.73 mm). There was a statistically significant difference in postprocedural ONSD between patients with and without PDPH development at T2 (p < 0.001) (Fig. 2).

**Fig. 4:**
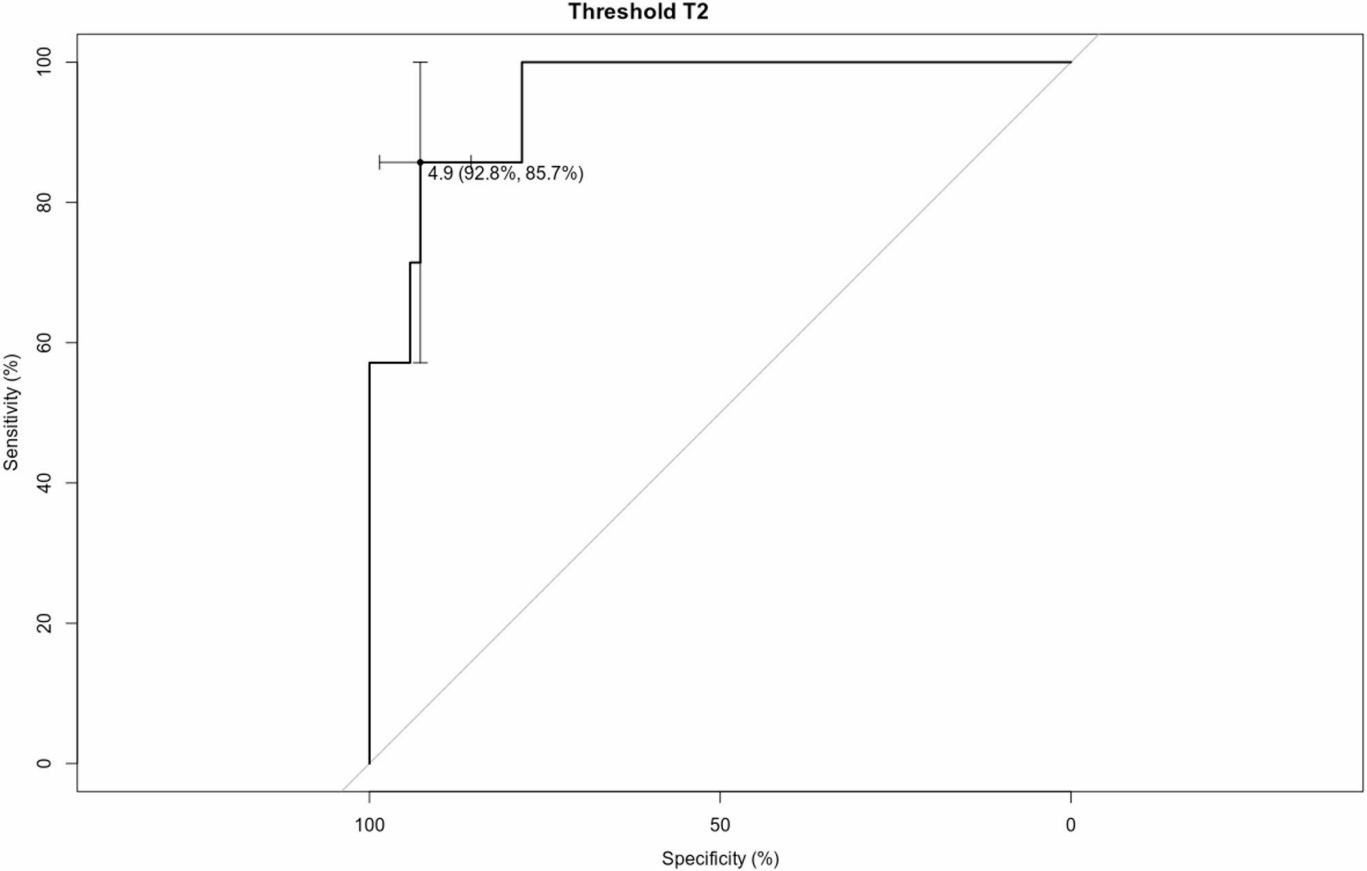
The ROC curve of the ONSD cutoff for PDPH prediction. ONSD T2 cutoff was 4.9 mm; AUC = 0.92, 95% CI 0.89 to 1.01, and p < 0.05*, T2 AUC: 0.93, sensitivity 85.7%, specificity 92.8%.

The ROC curve analysis showed the optimal ONSD cutoff value at T2 of 4.9 mm for predicting PDPH (AUC 0.92, 95% CI: 0.89 to 1.01). Adopting this cutoff value, the sensitivity and specificity were 85.7% and 92.8%, respectively (Fig. 4).

Based on multivariate logistic-regression models, Supplemental Table 1 displays a consistent decreasing effect of age on PDPH risk (OR ≈ 0.23, 95% CI 0.07–0.73, p ≈ 0.01–0.02 across all ONSD time points). A larger CSF withdrawal volume tends to increase PDPH risk (OR ≈ 1.78, 95% CI 0.69–4.61, p = 0.235), corresponding to an odds-ratio increase of about 10% per standardized unit (OR≈ 1.11, p = 0.08 in the original unscaled analysis). ONSD measured at 24 h (T2) is strongly inversely associated with PDPH (OR = 0.003, 95% CI 0.000–0.506, p = 0.026), indicating a dramatic reduction in odds when ONSD is higher at this time point. Baseline (T0) and immediate post-procedure (T1) ONSD do not reach significance (p > 0.4). Sex, needle gauge, chronic-headache history, and BMI remain non-significant in every model (all p > 0.27, with ORs close to 1 and wide confidence intervals).

## Discussion

This study shows that, induced by diagnostic LP, the ONSD decreases immediately and significantly, also confirming in accordance to other studies that the ONSD reacts real-time to changes in ICP. This study further confirms that such a noninvasive technique can be a tool for dynamic estimation and real-time monitoring of ICP, especially in an acute phase of change of ICP^7,16^.

The percentage of patients who developed PDPH was about 9% in our cohort. All 76 patients included showed a physiological reduction in ONSD after diagnostic LP. In the PDPH patients in particular, the decrease in ONSD was significant at 24 h after LP (T2).

Our results are similar to those of other recent studies, showing that assessing ONSD can predict PDPH^7,8,12^; however, with a different incidence, most likely due to a different study design, different needle sizes used and differences in the definition of the cut-off for ONSD in PDPH. The studies cited involved patients who underwent epidural analgesia for caesarean section or other operations^6–8^. Moreover, not all studies specify whether the measurement of ONSD adhered to recently published international guidelines^1^. Furthermore except in two studies, no measurement of ONSD is documented by screen capture^2,3^.

Our second significant and relevant finding is that patients in the PDPH group were markedly younger. Although not statistically significant, the proportion of women in the group of PDPH patients was higher than in the control group. This aspect is similar and is one well-documented risk factor for developing PDPH^17^.

We should consider that in our study, the decrease of ONSD after 24 h from LP (T2) was significant only in the group of PDPH, which showed a tendency to decrease furthermore after 48 h. According to our acquired data, the best ONSD cut-off value indicating decreased ICP was 4.9 mm, with 85.7% sensitivity and 92.8% specificity after 24 h.

It should be acknowledged that some patients without any particular complaints were discharged after 24 h. Therefore, we do not know whether all patients would also have presented a decrease in ONSD.

Accordingly, the postprocedural 24-h ONSD measurement can be used in combination with preprocedural measurement for PDPH prediction, especially in high-risk patients such as young women and those with reduced BMI. At first glance, it seems unrealistic to perform an ONSD measurement before every LP. On the other hand, this would be a non-invasive way of addressing the issue of increased ICP. In this perspective, the idea of general ONSD measurement before an LP is not too far-fetched. There is also promising data for the effective measurement of ONSD with automatic measurements and artificial intelligence methods^18^, which could extend such measurements serially and in an easy way for at-risk patients.

It identifies a cut-off of 4.9 mm, which should be viewed as a hypothesis for patients who develop post-dural puncture headache who underwent a lumbar puncture for diagnostic purposes.

The identification of such patients presenting with symptoms attributable to intracranial hypotension, combined with measurement of ONSD in both supine and orthostatic positions, could be a protocol to be applied in all subjects at risk^4,9^.

Furthermore, in contrast to other studies, we found a significant decrease in ONSD after 24 h, whereas a Chinese study found a decrease in ONSD of only 0.1 mm after LP; a value that is at the lower limit of the technique’s resolution^11^.

Finally, US-ONSD appears to be more suitable than other techniques, as it has the following advantages: low cost, short examination times, good reproducibility, bedside availability, non-invasiveness and simplicity of performance.

ONSD ultrasound may select those patients, who present symptoms compatible with PDPH. Serial ONSD measurements could objectively track recovery from post-lumbar puncture hypotension following conservative management or an epidural blood patch^19^.

In contrast, CT with cisternography has radiation exposure and low spatial resolution and MRI should only be reserved for severe cases with protracted symptoms to localise and quantify CSF leakage^20^.

It should be noticed that to date there is no clear cut-off value indicating CSF hypotension in the literature. The ONSD must be associated with compatible clinical symptoms. Another important area of application is to verify a normalisation of values in subjects where a patch has been applied or in subjects with protracted symptoms^19^.

### Limitations

This study has some limitations and strengths. First, this study was performed at a single center and achieved only a small group size of seven cases of PDPH. Moreover, the final sample size was slightly below the initially calculated target, we believe that this minimal shortfall is unlikely to have materially affected the study’s conclusions.

Second, we did not directly compare the ONSD measurement with CSF pressure. Nevertheless, measuring CSF opening pressure in the lateral decubitus position and correlating it with ONSD would have been desirable in future studies in a more appropriate setting. Third, the method is operator-dependent, and the experience of the neurosonologist measuring could influence the measurements of ONSD. Moreover, we were not able to measure the puncture speed and the number of attempts. Still, we standardised the technique in this case before starting the experiment. The cut-off of the normal value of the ONSD may vary anatomically from person to person. Moreover, the lateral resolution of TOS is around 0.4 mm, which makes the difference between pathological and non-pathological close and subject to possible error.

Finally, we defined the diagnosis of PDPH based on symptoms reported by the patient.

## Conclusion

In this study, we found that a decrease in ONSD at 24 h may suggest the onset of PDPH in some patients. Because we only detected an inferior rate of patients with PDPH than expected from earlier power calculation, these findings should be validated in further independent, multicenter studies. Additionally, developing an automated method for measuring ONSD could help identify these patients more effectively.

## Supplementary Information

Supplementary Information.

## Data Availability

The datasets used and analysed during the current study are available from the corresponding author on reasonable request.

## Abbreviations

BMI: Body mass index
CSF: Cerebrospinal fluid
CT: Computed tomography
ICP: Intracranial pressure
IIH: Idiopathic intracranial hypertension
LP: Lumbar puncture
MRI: Magnetic resonance imaging
NRS: Numerical rating scale
ONS: Optic nerve sheath
ONSD: Optic nerve sheath diameter
PDPH: Postdural puncture headache
ROC: Receiver operating characteristic
SD: Standard deviation
SIH: Spontaneous intracranial hypotension
TOS: Transorbital sonography
US-ONSD: Ultrasound-guided optic nerve sheath diameter
