# Supplementary material for: Ultrasound measurement of optic nerve sheath diameter pre and post lumbar puncture for prediction of postdural puncture headache
Source: Sci Rep. 2026 Feb 20;16:7468. doi: 10.1038/s41598-026-40311-1 (PMC12929585; doi:10.1038/s41598-026-40311-1)
Supplement: Supplementary file 1 — Supplementary Information. [file 41598_2026_40311_MOESM1_ESM.pdf]

Covariates	OR (B)	95 %-CI (B)	%-Change (B)	p (B)	OR (ONSDT0M)	95 %-CI (ONSDT0M)	%-Change (ONSDT0M)	p (ONSDT0M)	OR (ONSDT1M)	95 %-CI (ONSDT1M)	%-Change (ONSDT1M)	p (ONSDT1M)	OR (ONSDT2M)	95 %-CI (ONSDT2M)	%-Change (ONSDT2M)	p (ONSDT2M)
**Intercept**	0.051	0.009 – 0.283	-94.9 %	0.0007	1.187	0.000 – 35870.85	+18.7 %	0.974	0.279	0.000 – 5367.98	-72.1 %	0.800	8.55×10^{10}	$4.92 – 1.49 \times 10^{21}$	+8 553 200 %	0.036
**Sex (m)**	0.436	0.059 – 3.216	-56.4 %	0.4153	0.518	0.064 – 4.173	-48.2 %	0.536	0.461	0.060 – 3.525	-53.9 %	0.455	7.49	$0.185 – 3.03 \times 10^{2}$	+6 488 %	0.286
**Age**	0.229	0.072 – 0.728	-77.1 %	0.0125	0.230	0.073 – 0.729	-77.0 %	0.0125	0.230	0.073 – 0.728	-77.0 %	0.0124	0.349	0.070 – 1.746	-65.1 %	0.200
**Depleted CSF**	1.782	0.688 – 4.610	+78.1 %	0.235	1.740	0.670 – 4.519	+74.0 %	0.255	1.765	0.682 – 4.570	+76.5 %	0.242	1.44	0.543 – 3.80	+44.0 %	0.466
**Gauge**	1.249	0.610 – 2.558	+24.9 %	0.543	1.283	0.623 – 2.645	+28.3 %	0.499	1.272	0.618 – 2.619	+27.2 %	0.513	1.34	0.477 – 3.77	+34.1 %	0.578
**History of chronic headache (ja)**	1.502	0.201 – 11.249	+50.2 %	0.692	1.529	0.201 – 11.613	+52.9 %	0.681	1.467	0.195 – 11.056	+46.7 %	0.710	0.753	0.027 – 21.18	-24.7 %	0.868
**BMI (z)**	0.632	0.205 – 1.944	-36.8 %	0.423	0.606	0.195 – 1.883	-39.4 %	0.387	0.621	0.201 – 1.918	-37.9 %	0.408	0.398	0.076 – 2.089	-60.2 %	0.276
**ONSDT0M**	–	–	–	–	0.556	0.083 – 3.736	-44.4 %	0.546	–	–	–	–	–	–	–	–
**ONSDT1M**	–	–	–	–	–	–	–	–	0.707	0.098 – 5.119	-29.3 %	0.731	–	–	–	–
**ONSDT2M**	–	–	–	–	–	–	–	–	–	–	–	–	0.003	0.000 – 0.506	–99.7 %	0.026

Supplemental Table 1: Multivariate logistic regression analysis of post dural puncture headache (PDPH) using four scaled generalized linear models. Model 1 (baseline) incorporates sex (male), age (z score), CSF withdrawal volume (z score), needle gauge (z score), history of chronic headache (yes), body mass index (z score). Model 2 includes ONSD before lumbar punction. Model 2 substitutes ONSD with the ONSD measured 1 h after the procedure (T1 M); Model 3 substitutes ONSD with the ONSD measured 24 h after the procedure (T2 M); For each predictor the table lists the odds ratio (OR), the 95 % confidence interval for the OR, the percent change in odds relative to the reference ((OR − 1) × 100), and the Wald test p value. Continuous variables were centered and scaled (z scores) prior to model fitting.

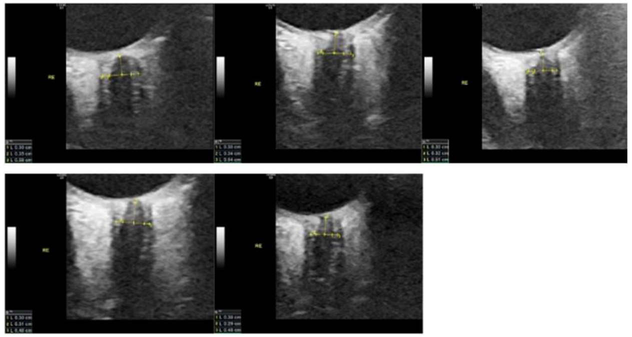

Supplemental Figure 1: Illustration of the change of optic nerve sheath diameter (ONSD) in a patient with post-dural puncture headache (PDPH). ONSD image from transorbital ultrasound. Progressive reduction of the diameter of the ONSD in a symptomatic patient that occurs from T0 (before LP, top left), 5.8 mm, to T4, 4.8 mm (72 hours after LP, bottom right). The yellow arrow (3 measures of each figure) indicates the subarachnoid space around the optic nerve measured by ultrasound. ONSD was assessed 3 mm behind the papilla in axial plane on both sides using a perpendicular axis. The field of view is adjusted to a depth of 40 mm (For better Illustration, the image's zoom was chosen).
